# Monocytes are increased in pregnancy after gestational hypertensive disease

**DOI:** 10.1038/s41598-022-13606-2

**Published:** 2022-06-20

**Authors:** James S. Castleman, Gregory Y. H. Lip, Eduard Shantsila

**Affiliations:** 1 West Midlands Fetal Medicine Centre, Birmingham Women’s and Children’s Hospital NHS Foundation Trust, Birmingham, UK; 2 Liverpool Centre for Cardiovascular Science, Institute of Ageing and Chronic Disease, University of Liverpool, Liverpool, UK; 3 Aalborg Thrombosis Research Unit, Department of Clinical Medicine, Aalborg University, Aalborg, Denmark; 4 Institute of Population and Health Sciences, University of Liverpool, Liverpool, UK

**Keywords:** Monocytes and macrophages, Prognostic markers

## Abstract

Monocytes derive from bone marrow and circulate in the blood. They phagocytose, produce cytokines and present antigens. Individual monocyte subsets play distinct roles in the pathogenesis of cardiovascular disease, but their implications in gestational hypertensive disease are unclear. Our objective was to examine the difference in monocyte subsets between pregnant women with or without previous hypertension in pregnancy. Women were enrolled in a prospective observational study in which monoclonal antibodies against cell surface receptors were used to detect monocytes in the peripheral blood by flow cytometry. We compared 17 pregnant women with previous hypertension in pregnancy (Group 1) and 42 pregnant women without previous gestational hypertensive disease (Group 2) with 27 healthy, non-pregnant controls (Group 3). The pregnant women were studied at 13 ± 1 weeks gestation. Monocyte subsets were quantified by flow cytometry: Mon1 (CD14++CD16-CCR2+), Mon2 (CD14++CD16+CCR2+), Mon3 (CD14+CD16+CCR2-), their aggregates with platelets and expression of the surface markers. The groups were well-matched for age, body mass index and ethnicity (*P* > 0.05 for all). Mon1 counts were higher in women with a history of gestational hypertension or preeclampsia compared to other groups (Group 1 = 441 per *µl* (376–512); Group 2 = 357 (309–457); Group 3 = 323 (277–397); *P* < 0.001). Mon3 was higher in both groups of pregnant women compared to non-pregnant controls (Group 1 = 51 (38–62); Group 2 = 38 (29–58); Group 3 = 26 (20–40), *P* = 0.002). Increased monocytes in women with a previous hypertensive pregnancy generates a hypothesis that these cells may link hypertension in pregnancy, chronic inflammation and future cardiovascular risk.

## Introduction

Hypertension is seen in up to 10% of pregnancies^1,2^. It includes chronic hypertension, gestational (pregnancy-induced) hypertension (GH) and preeclampsia (PE), and accounts for approximately 14% of maternal deaths^3^ and is a risk factor for future cardiovascular disease^4^. Gestational hypertension is also an important cause of fetal morbidity and mortality, mostly due to growth restriction and preterm birth^1^.

Mechanisms of hypertension in pregnancy and its complications are still insufficiently understood. Hypertension in pregnancy, preeclampsia and atherosclerotic cardiovascular disorders share many of the same risk factors and underlying pathophysiology, such as endothelial dysfunction, vascular inflammation and remodelling^5,6^. Monocyte subsets are implicated in the pathophysiology of cardiovascular disease and may play a role in the pathogenesis of pregnancy hypertension.

Monocytes make up 3–8% of peripheral blood leucocytes and play major role in phagocytosis and innate immunity^7,8^. They are also involved in procoagulant states through their interaction with platelets and play a part in regeneration after tissue injury^9^. Monocytes are the largest pool of progenitor cells, able to differentiate into many cells types^10,11^. Differentiation of monocytes in various cellular milieu allows them to perform tissue-specific roles in different parts of the body^12^. Monocyte trafficking allows them to migrate out of the circulation, where their half-life is around three days^13^, into areas of injury where they are involved in resolving inflammation and restoring the tissue and releasing cytokines^8,14^.

Monocytes in the peripheral blood can be divided into three types based on their expression of cell surface receptors^7,11^. Different monocyte subsets have different biological functions and play distinct roles in pathogenesis of multiple cardiovascular and inflammatory disorders^15–17^.

Platelets are activated when there is denudation of the endothelium, and if they undergo degranulation and adhere to circulating monocytes, then monocyte-platelet aggregates (MPAs) are formed^7^. Platelets have a role in regulation of circulating cell types and their physical interaction with monocytes is an example of such an interaction that may influence their function^18,19^. It is proposed that the cross-talk between monocytes and platelets is the link between thrombosis and inflammation^20^. Monocyte-platelet aggregates in peripheral blood have been shown to correlate directly with blood pressure and are particularly closely related to systolic blood pressure^21^.

Functionally distinct monocyte subsets play different roles in the pathogenesis of cardiovascular disease^22^, but their implications in hypertension in pregnancy are unclear. Women with prior hypertension in pregnancy have altered cardiovascular adaptation to pregnancy, which may be related to increased proinflammatory monocytes and lead to the development of recurrent hypertension. We aimed to investigate monocyte heterogeneity in high risk pregnancies in women with previous gestational hypertensive disease.

## Methods

### Study populations

The Evaluating Cardiovascular Changes in Hypertension in Obstetrics (ECCHO) study was a prospective observational study in which women were recruited at early pregnancy and studied throughout their gestation. This analysis of the ECCHO study compared count and phenotypic characteristics of monocyte subsets between 17 pregnant women with gestational hypertension or preeclampsia in a previous pregnancy, 42 pregnant women with no history of hypertension, and 27 healthy non-pregnant women. The diagnosis of the hypertensive disorders in pregnancy was in accordance with the guidelines of the National Institute for Health and Care Excellence (sustained elevation in blood pressure greater than 140/90 mmHg after 20 weeks of gestation, with or without significant proteinuria) confirmed by case note review^1^. Women with chronic hypertension were excluded.

Women were recruited at City Hospital, Birmingham. The pregnant women were studied at 13 ± 1 weeks of gestation. Blood sampled from a peripheral vein was promptly processed and analysed by flow cytometry. This study was approved by the Research and Development Department of Sandwell and West Birmingham Hospitals NHS Trust (Reference 13CARD65, 27/11/13), following review by the institution’s Ethics Committee. All participants gave written informed consent. The study was conducted in accordance with the Declaration of Helsinki.

### Blood pressure measurement

Blood pressure was measured with a digital blood pressure monitor (Omron Corporation, Tokyo, Japan). This automated, electronic, oscillometric device is validated for use in pregnancy^23^ and was calibrated throughout the study. The brachial artery of the non-dominant arm was used for the blood pressure recording. Care was taken to ensure that the arm was free of clothing and that appropriate cuff size was used depending on mid-arm circumference. Blood pressure was recorded whilst the patient was seated comfortably and silently, with the arm supported at the level of the heart. The woman was asked to sit upright and still, with her back well supported, legs uncrossed and feet flat on the floor. Three readings were taken one minute apart and the mean average was calculated.

### Flow cytometry

A FACSCalibur flow cytometer (Becton Dickinson [BD], Oxford, UK) was used for monocyte quantification and characterisation. A ‘Mastermix’ of four mouse anti-human monoclonal fluorochrome conjugated antibodies was made in small batches. The antibodies used were anti-CD16-Alexa Fluor 488 (clone DJ130c, Bio-Rad Laboratories, Oxford, UK), anti-CD14-PE (clone MфP9, BD), anti-CD42a-PerCP (clone Beb1, BD) and anti-CCR2-APC (clone 48607, R&D Systems, Abingdon, UK). A 12.5 μL volume of Mastermix (including anti-CD14-PE 2.5μL; anti-CD16 2.5μL; anti-CD42a-PerCP 5μL and anti-CCR2-APC 2.5μL) was added to a TruCount tube (BD, Oxford, UK) containing a precise number of fluorescent count beads. To this tube, 50 μL of EDTA-anticoagulated whole blood (from 13 ± 1 weeks gestation for the pregnant women) was added. The TruCount tube was then incubated for 15 min. Lysis of the red blood cells was achieved using 450 μL FACS Lysing solution (BD) and a further 15 min incubation. The sample was diluted with 1500 μL of phosphate buffered saline prior to immediate flow cytometric analysis. Appropriate isotype controls were used to define CD14, CD16 and CCR2 and monocyte subsets were defined as CD14++CD16-CCR2+(Mon1), CD14++CD16+CCR2+(Mon2) and CD14+CD16++CCR2-(Mon3)^7^. Time delay between sample collection and processing was always less than one hour, as delays in processing can compromise results^24^. The absolute monocyte count (cells/μL) was calculated from the total number of monocytes proportional to the number of TruCount beads. Each subset could be counted individually according to the gating strategy.

Detection of monocyte-platelet aggregates (MPAs) was achieved by their CD42a positivity. CD42a (glycoprotein IX) is a platelet marker and MPA events were positive for monocyte and platelet markers. The MPAs associated with each monocyte subset, and the total number of MPAs, were expressed as a proportion of the number of TruCount beads as described above. Thus, MPAs associated with Mon1 (MPA1) are CD14++CD16-CCR2+CD42a+, MPAs associated with Mon2 (MPA2) are CD14++CD16+CCR2+CD42a+ and those MPAs associated with Mon3 (MPA3) are CD14+CD16++CCR2-CD42a++.

### Statistical analysis

ANOVA was used to assess differences between the three study groups for normally distributed data. The Kruskal–Wallis test was used to analyse non-normally distributed data. Post hoc testing (Dunn-Bonferroni procedure) was performed to account for comparisons between multiple groups, and differences were considered significant if *P* < 0.01 to account for multiple testing. The Mann–Whitney test was applied to comparisons of non-normally distributed data between the two groups of pregnant women. The statistical analysis was performed using Stata version 14 (StataCorp, TX, www.stata.com).

### Ethics approval

This study was approved by the Research and Development Department of Sandwell and West Birmingham Hospitals NHS Trust (Reference 13CARD65, 27/11/13), following review by the institution’s Ethics Committee.

### Consent to participate

All participants gave written informed consent. The study was conducted in accordance with the Declaration of Helsinki.

### Consent for publication

Provided by all authors.

## Results

Women in the 3 groups were matched for age, ethnicity and smoking status (*P* > 0.05 for all, Table 1). Aspirin use was higher in pregnant women with previous hypertension (*P* < 0.001 vs other groups). This group had a trend towards higher body mass index (*P* = 0.07) and had higher blood pressures (*P* = 0.001 for mean arterial pressure). Heart rate was higher and haemoglobin lower in the pregnant women (*P* < 0.001 vs non-pregnant controls). White cell count was significantly higher in pregnancy, and higher still in pregnant women with previous hypertension (*P* < 0.001).

**Table 1:** Demographic and clinical characteristics.

Characteristic	Pregnant with previous hypertension (n = 17)	Pregnant without previous hypertension (n = 42)	Non-pregnant controls (n = 27)	*P*
Age, years	30 (27–33)	30 (25–33)	27 (25–34)	0.68
**Parity, *n* (%)**				
Nulliparous	0 (0)	19 (45)	22 (81)	< 0.001
Parous	17 (100) *^†^	23 (55)^†^	5 (19)	
**Ethnicity, *n* (%)**				
White	6 (35)	16 (38)	18 (67)	0.28
South Asian	8 (47)	18 (43)	2 (7)	
Black	1 (6)	6 (14)	5 (19)	
East Asian	0 (0)	1 (2)	1 (4)	
Other/mixed	2 (12)	1 (2)	1 (4)	
Asthma, *n* (%)	2 (12)	1 (2)	3 (8)	0.34
Diabetes, *n* (%)	1 (6)	3 (7)	0	0.38
Aspirin, *n* (%)	6 (35)	1 (2)	0 (0)	< 0.001
Hormonal contraception, *n* (%)	0^†^	0^†^	10 (37)	< 0.001
Smoker, *n* (%)	2 (12)	2 (5)	1 (4)	0.50
BMI, kg/m^2^	28 (26–30)	26 (22–29)	24 (21–29)	0.07
**Peripheral BP, mmHg**				
Systolic	117 (113–121)*	108 (100–113)	113 (101–122)	0.006
Diastolic	72 (66–75)*	64 (58–67)	66 (59–71)	0.007
Mean	87 (82–92)*	77 (72–81)	81 (73–86)	0.001
**Central BP, mmHg**				
Systolic	101 (99–105)*	92 (86–96)	98 (89–102)	0.001
Diastolic	72 (68–77)*	65 (59–69)	67 (59–72)	0.004
Mean	86 (80–92)*	77 (72–81)	81 (73–86)	0.01
Heart rate, beats per minute	82 ± 10*^†^	74 ± 9^†^	65 ± 11	< 0.001
Haemoglobin, g/L	118 ± 12^†^	120 ± 9^†^	132 ± 11	< 0.001
White cell count, × 10^9^/L	10.6 (9.3–11.6)*^†^	8.3 (7.1–9.2)^†^	6.3 (5.3–7.1)	< 0.001
Platelets, × 10^9^/L	259 (226–306)	241 (199–289)	270 (249–303)	0.15
Creatinine, µmol/L	51 (49–54)^†^	53 (50–55)^†^	66 (64–71)	< 0.001

*BMI* body mass index, *BP* blood pressure.

Continuous data are expressed as median (interquartile range). Categorical data are expressed as *n* (%).

*P < 0.01 versus pregnant without previous hypertension; ^†^*P* < 0.01 versus non-pregnant controls.

**Figure 1:**
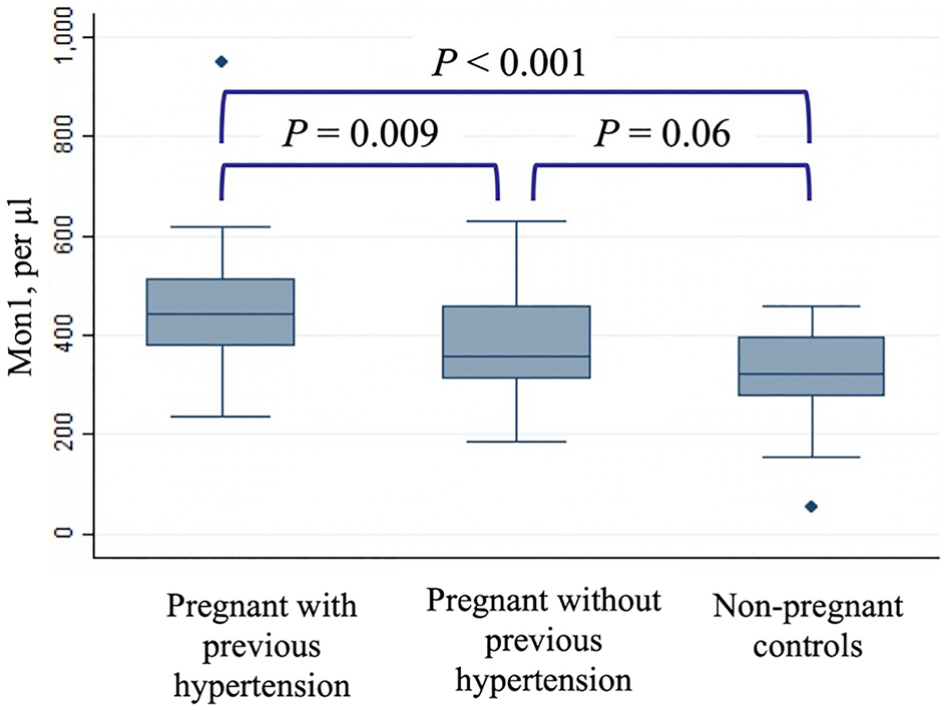
Mon1 counts in the study groups. The figures shows increased counts of Mon1 subset in pregnant women with previous hypertension. Mon1, CD14++CD16-CCR2+.

Mon1 count was significantly higher in pregnant women with previous hypertension compared to pregnant women without previous hypertension (*P* = 0.009) and non-pregnant controls (*P* < 0.001, Fig. 1, Table 2). The Mon1 count in pregnant women without previous hypertension was not significantly higher than in non-pregnant controls (*P* = 0.06). Mon3 count was increased in both groups of pregnant women vs. non-pregnant controls (*P* = 0.002 for the group with previous hypertension; *P* = 0.007 for the group without previous hypertension). The increase in total monocytes in women with prior hypertension was highly significant compared to non-pregnant controls (*P* < 0.001) and trended towards significance compared to the pregnant women without hypertension (*P* = 0.053).

**Table 2:** Monocyte characteristics.

Characteristic	Pregnant with previous hypertension (n = 17)	Pregnant without previous hypertension (n = 42)	Non-pregnant controls (n = 27)	*P*
**Monocyte counts, per µl**				
Total	545 (455–592)^†^	425 (374–514)	378 (293–463)	< 0.001
Mon1	441 (376–512)*^†^	357 (309–457)	323 (277–397)	< 0.001
Mon2	15 (9–49)	19 (10–41)	22 (10–41)	0.99
Mon3	51 (38–62)^†^	38 (29–58)^†^	26 (20–40)	0.002
**Monocyte-platelet aggregates, per µl**				
Total	51 (43–69)^†^	47 (34–66)	41 (26–46)	0.01
Associated with Mon1	40 (33–62)^†^	35 (26–47)	32 (23–38)	0.02
Associated with Mon2	2 (1–6)	3 (1–6)	4 (2–5)	0.99
Associated with Mon3	6 (4–8)^†^	5 (4–9)^†^	3 (2–5)	0.002
**Monocyte CCR2 expression, MFI**				
Mon1	113 (88–140) ^†^	126 (98–147)^†^	167 (117–203)	0.002
Mon2	96 (86–112)	91 (78–121)^†^	121 (92–151)	0.037
Mon3	17 (16–19)	16 (15–18)	18 (16–19)	0.14
**Monocyte CD14 expression, MFI**				
Mon1	1586 (1233–1712)	1535 (1408–1729)	1651 (1359–1766)	0.68
Mon2	1199 (887–1612)	1396 (1146–1684)	1566 (1189–1769)	0.23
Mon3	200 (165–217)	183 (136–228)	198 (153–235)	0.91
**Monocyte CD16 expression, MFI**				
Mon2	91 (84–120)	91 (77–111)	90 (81–95)	0.60
Mon3	185 (166–237)	215 (172–272)	236 (144–292)	0.39
**CCR2 expression on monocyte-platelet aggregates, MFI**				
Associated with Mon1	111 (85–126)^†^	111 (85–137)^†^	148 (111–193)	< 0.001
Associated with Mon2	123 (96–142)	107 (95–142)	133 (102–159)	0.44
Associated with Mon3	17 (14–17)	17 (15–19)	17 (16–19)	0.17
**CD42a expression on monocyte-platelet aggregates, MFI**				
Associated with Mon1	46 (43–48)	43 (40–48)	42 (39–49)	0.42
Associated with Mon2	57 (48–100)	56 (48–71)	51 (47–60)	0.19
Associated with Mon3	46 (44–51)	48 (42–55)	49 (43–58)	0.95

*MFI* mean fluorescent intensity.

Data expressed as median (interquartile range).

*P < 0.01 versus pregnant without previous hypertension; ^†^*P* < 0.01 versus non-pregnant controls.

MPA1 were increased in pregnant women with previous hypertension (*P* = 0.006 vs. the non-pregnant control group). MPA3 were increased in both groups of pregnant women (*P* ≤ 0.005 for both). For total monocyte platelet aggregate count, the only significant difference observed on cross sectional comparison at baseline was a higher count in pregnant women with previous hypertension compared to non-pregnant controls (*P* = 0.004). There was no difference in Mon2 and MPA2 between the groups (*P* > 0.05). CCR2 expression was reduced on both Mon1 and MPA1 in pregnant women groups (*P* < 0.005 vs. controls for both).

The study was not powered to assess a difference in the incidence of hypertension between the groups of pregnant women. All women had normal blood pressure at 3 months postnatal.

## Discussion

The study shows for the first time increased counts of monocytes (predominantly the Mon1 subset) and their aggregates with platelets, in pregnant women previously affected by a hypertensive disorder in pregnancy, compared to pregnant women without prior hypertension. The associations described may be involved in the aetiology of hypertension in pregnancy and its conferring increased cardiovascular risk after an affected pregnancy. Whilst it is scientifically plausible that the associations could imply causation, they could also represent coincidental changes, or changes associated with compensatory mechanisms for other disease-related changes. The overall increase in monocyte count in pregnancy is consistent with previous studies^25^ demonstrating an immunological response to the gravid state.

Mon1 are large cells, which are highly phagocytic and actively recruited to sites of inflammation^26^. Mon1 phagocytose low-density lipoprotein and generate reactive oxygen species^27^. This subset expresses genes involved in angiogenesis, wound healing and coagulation^28^. Mon2 expresses genes associated with inflammation and angiogenesis, which the other subsets do not^29^. They also have cell surface receptors linked with angiogenesis and tissue repair and remodelling^10,18,30^. Like Mon1, Mon2 have been shown to produce reactive oxygen species^29^. Mon3 may have a protective function in tissues with a patrolling and reparative role in case of injury. They are smaller and less granular than Mon2 and have a much lower phagocytic activity than Mon1 and Mon2^7,10^. If there is endothelial dysfunction Mon3 can migrate out of the circulation^30^.

The increase in Mon1 counts shown in pregnant women with previous hypertension could be interpreted in several ways. The increased number of these circulating monocytes which release cytokines could be involved in the pathogenesis of prehypertension and hypertension in pregnancy. Endothelial damage/dysfunction and resulting inflammatory response may be responsible for the increased monocyte counts. The increase MPA, especially those associated with Mon1, in women with prior hypertension reflects monocyte activation in these women. Previous work has shown Mon2 to be increased, and make up a greater proportion of the monocyte count in women with preeclampsia, with counts correlating with disease severity^25^. Our data show Mon2 to be unchanged at baseline or during pregnancy in women with prior gestational hypertensive disease. The increase in Mon3 in both groups of pregnant women could point to a pattern of increased immune surveillance. It is possible that pregnant women with previous hypertension have an increased inflammatory response during the next pregnancy. It could also be true that having had a pregnancy complication last time, the innate immune system shows increased activity in the subsequent pregnancy.

Monocyte migration can be controlled by the modulation of their cell surface receptors. Monocyte chemo attractant protein-1 (MCP1) binds to CCR2 on Mon1 to promote its active recruitment to sites of inflammation^26^. Bosco et al. propose that downregulation of CCR2 leads to trapping of monocytes to retain them at pathological sites. A reduction in CCR2 expression has been shown in hypoxic states^31^. Increased expression of CCR2 reflects increased activation of monocytes. CCR2 being less expressed on both subsets on which it is present (Mon1 and Mon2) in pregnant women compared to non-pregnant controls may mean it is attenuated in pregnancy as part of the immunological adaptation to the pregnant state. Reduced CCR2 expression may be detrimental if it results in less clearance of cellular debris and reduced beneficial phagocytosis and cytokine release. In contrast, CCR2 expression has been shown to be increased on monocytes of obese women, with enhanced migration towards inflammatory stimuli^32^.

Evidence has accumulated over the last several years for the distinct subpopulations of monocytes with distinct roles in the pathogenesis of cardiovascular diseases, which are often characterised by tissue injury and inflammation. Whilst the primary role of monocytes is protective (neutralisation and elimination of pathogens via cytokine production and phagocytosis) they also have a detrimental role causing injury. When monocytes are high in number or activity, tissue damage may result in adverse outcomes^7^. For example shear stress and adherence and infiltration of monocytes in endothelial injury contributes to atherosclerosis^33^. In pregnancy, blood monocytes are increased as part of the innate immune system’s adaptation^25^. Mon1 has been shown to comprise a lower percentage of total monocytes in pregnant women, with a greater reduction in women with preeclampsia, attributed to an increase in Mon2^25^. TLR4 up regulation has been demonstrated in Mon3 and neutrophils in women with preeclampsia^34^. Preeclampsia has classically been considered a disease of placental aetiology and macrophages play an important role at the fetomaternal interface^35^. Macrophages are involved in immune tolerance and defence, in placentation and in parturition^35^. Melgert and colleagues describe the infiltration of the uterine decidua by monocytes, which become macrophages and dendritic cells involved in spiral artery remodelling and immune tolerance of the “semi-allogenic” fetus^25^. Platelets are activated in preeclampsia and activated platelets lead to an increase in Mon2. These are possible mechanisms for the increased proportion of CD16+ monocytes have been shown to be increased in preeclampsia^25^. Mon3 has also been shown to be elevated in preeclamptic women^34^.

## Strengths and limitations

Studies with a small sample size are more susceptible to random errors. The care taken during data acquisition, checking and analysis should minimise this risk. The small number of participants in the study increases the chance of a false negative findings, the so-called Type 2 error. In order to reduce the risk of false positive findings, post hoc testing was employed in order to raise the threshold for significance levels as previously described. Because of the paucity of existing literature on the roles of the monocyte subsets in pregnancy, the potential for confounding is greater. We measured monocytes in peripheral blood, and these levels might not reflect the counts of their counterparts in the tissues, such as macrophages in the placenta or myocardium.

Differences between the groups (and indeed aspects in which they are similar) could be attributable to variables which are not controlled for. An example is aspirin use in the study group. It was not possible to exclude women taking aspirin as aspirin is recommended for all women with previous hypertension in pregnancy. Antiplatelet therapy has been previously shown to reduce MPAs^36,37^. Recent evidence from a study which assured compliance with aspirin therapy showed no significant difference in MPA count with low dose aspirin^38^. In this study MPAs were higher pregnant women with previous hypertension in whom aspirin consumption was higher. There was no difference in platelet count between the groups. Sensitivity to aspirin and treatment response varies^39^. Patients whose platelet reactivity remains high despite antiplatelet treatment are at increased risk of major adverse cardiac events and this might be related to the pro-inflammatory effects of MPA formation^40^. It is likely that other factors in pregnant women with previous hypertension cause platelet activation and their complex formation with monocytes despite being on treatment. The fact that all women with previous pregnancy hypertension were by definition parous introduced another difference between the groups. Matching for parity would have necessitated the exclusion of primigravidae from the study. Restricting recruitment to multiparous women would have reduced rate of recruitment and the ability to compare findings with other published work. The lack of preconceptual and postnatal assessment prohibits the analysis of trends in Mon1 count. Longitudinal data would enhance future studies of monocyte subsets in pregnancy.

## Conclusions

We have described for the first time changes in monocyte subsets pregnant women with previous gestational hypertensive disease. These preliminary data demonstrate the importance of separate analysis of the three distinct monocyte subsets for understanding of implication of monocytes in processes associated with hypertension in pregnancy. Possible (patho)physiological and clinical effects of the changes in monocytes subsets in pregnancy will need to be established in the future.

## Data Availability

At the time of participant consent there was not provision for external data sharing.
